# Stomach and Liver

**Published:** 1876-05

**Authors:** 


### STOMACH AND LIVER.

My Dear Doctor:—When I wrote my last letter to you about a certain lazy liver which sails as passenger in my ship, I had no idea that you would think the "weary-graph" worth printing. I observe a request in your excellent Journal for this month, from some poor bilious wight like myself, that the minister would give an account of the trotting-match between lazy liver and nimble stomach. I fear I shall fail to do justice to the subject, and only write, my dear Doctor, on condition that, if the manuscript will not bear your editorial criticism, you will do me and your readers the favor to hand it to Biddy, to light the kitchen-fire.

We are upon the turf with our two nags—Liver and Stomach—and you may appoint any one you please on the intestinal race-course to hold the stakes. Just take a look at the animals. They have run together a thousand times before, and "Stomach" has won the day almost every time. He is a flat-footed creature, high-mettled, nervous, irritable, as dyspeptics' stomachs always are. Poor "Liver" is a raw-boned, spavined thing, stands still, to be sure, on four legs, but in motion is a tripod, with the fourth leg in tow, as unpromising a bit of locomotive flesh as was ever put into harness.

I don't know as I would trust even yourself, Doctor, to drive "Liver." I know the nag better myself (*my* nag, I mean): so I am mounted, and we are off. "Liver" is a tricky creature. He sometimes gets a fit of laziness just when you want to use him. I have often had experience of this, when having given him a good "feed" over night, I have found him next morning as sleepy as a Chinaman. The only way to do anything with the fellow is, to put him to bed without his supper. "Liver" likewise has a habit of baulking on the most trivial occasion. I have sometimes got into a fret, or perhaps actually lost my temper, and the creature would stop right in the road, as motionless as Lot's wife. I have often started him again with a hearty laugh or a frolic with the children, and not long ago I made him quite nimble with an hour's reading of "Pickwick" after dinner. He must be humored. I never knew him fail to baulk when I lost my patience.

Another trait:—The nag is exceedingly capricious about the kind of load he carries. There are certain things which scientific folks call carbonaceous, or non-nitrogenous. Such are sugar, molasses, butter, and fat of all kinds. Just as sure as I compel "Liver" to drag much of these commodities, "Stomach" comes out a dozen lengths ahead. They are what the old Roman soldiers called *impedimenta*, nothing but *baggage*, and the less one has the better.

Another thing, "Liver" won't bear much whipping. He gallops on bravely for a little while under the lash, but sinks down exhausted in the road, and his rider must nurse him, and make him fast like a monk, and coax him like a spoiled child, before he will get up and be ready for another start. I once took three blue pills in a week by the advice of an empiric. My "Liver" went like Jehu or Mazeppa, for just seven days, and it was as many months almost before I could wake up the poor jaded thing to action again.

Now, my dear Doctor, perhaps you or your correspondent will say that I have not yet described the race. True, but have I not done something of more practical profit, which is to describe a few requisites to the art of successful driving. I'll tell you the result, and it ought to cheer every jaundice-eyed, sallow-faced dyspeptic beneath the sun, or above the ground. The nags came through "neck and neck," and the bowels being the judges, the spavined animal and the mettled steed were well mated. To conclude, the issue of the race depends not so much on having a *two-forty* nag, as upon the matter of skillful horsemanship. If ever a man had a slow liver, I have. It has done nothing but limp or lie down in the road for fifteen years, but even with these unpromising antecedents, I can make it do its daily work.

I know no better medicines for a torpid liver than the following: A thankful, happy heart, a light stomach, plenty of fresh air and exercise, and no medicine. To these might be added, early rising, which I practice with great advantage, having written nearly one-half of my last Sunday's sermon before breakfast on Saturday morning, and having to-day swallowed a good draught of theology, and a sparkling bumper of belles lettres, besides writing this scrawl, before I sat down to my wheaten-grits, and bade my wife good morning at the table.

Gratefully yours, LEONARD.

